# Leveraging artificial intelligence for risk stratification of inherited cardiomyopathies in under-resourced settings

**DOI:** 10.1016/j.hroo.2025.07.020

**Published:** 2025-08-05

**Authors:** Salah H. Alahwany, Omnia Kamel, Amir Abdelghany, Ahmed Ammar

**Affiliations:** 1 Arrhythmogenic Cardiomyopathy Program, Vanderbilt Heart and Vascular Institute, Nashville, Tennessee; 2 Cardiology Department, Ain Shams University, Cairo, Egypt; 3 Cardiology Department, Aswan Heart Center, Aswan, Egypt; 4 Cardiology Department, King Abdullah Medical Complex, Jeddah, Saudi Arabia; 5 Cardiology Department, Worcestershire Acute Hospitals NHS Trust, Worcester, United Kingdom

**Keywords:** Artificial intelligence, Deep learning, Machine learning, Inherited cardiomyopathy, Risk stratification, Sudden cardiac death, Ventricular arrhythmias, Under-resourced settings, Developing countries

## Abstract

Inherited cardiomyopathies are a significant global cause of sudden cardiac death, particularly among younger individuals and those in under-resourced regions. Despite progress in diagnostics and therapeutics, screening and risk stratification remain challenging due to genetic complexity, variable clinical presentation, and the interpretive limitations of current electrophysiological and imaging tools. Artificial intelligence (AI)—particularly machine learning, deep learning, and natural language processing offers transformative potential by enabling large-scale analysis of complex data and detecting subtle disease patterns which could potentially improve diagnostic accuracy and cost-effectiveness, particularly in low-resource environments. This review evaluates the limitations of existing risk models, synthesizes disease-specific AI applications within a unified framework, and explores the role of AI in advancing personalized care and risk prediction in underserved populations.

## Graphical abstract

**Figure:**
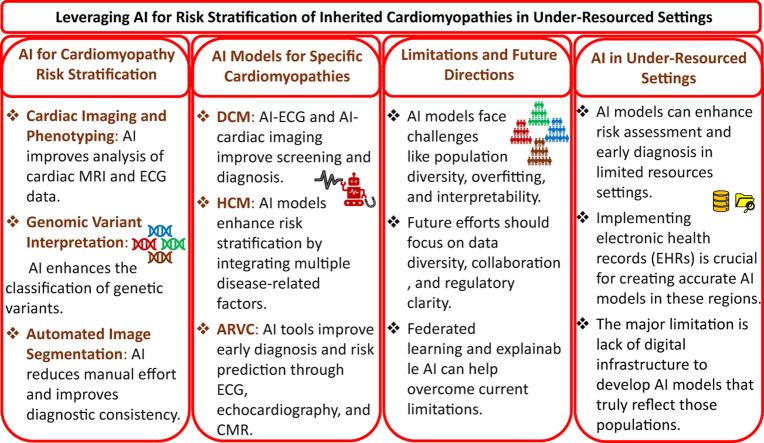

**Key Findings**

▪ Artificial intelligence (AI) enables scalable and cost-effective screening for cardiomyopathies in under-resourced settings by leveraging widely available tools like electrocardiograms, digital stethoscopes, and point-of-care ultrasound, reducing the reliance on advanced imaging and specialist interpretation.
▪ AI-driven models can improve early detection and risk stratification of inherited cardiomyopathies by automating the analysis of clinical and physiological data, and supporting diagnosis in primary care and community environments where access to traditional diagnostics is limited.
▪ Integrating AI into routine care helps overcome barriers to timely diagnosis, facilitates task-shifting to non-specialist health workers, and supports broader access to life-saving interventions in low-resource environments.

## Introduction

Inherited cardiomyopathies—including hypertrophic cardiomyopathy (HCM), dilated cardiomyopathy (DCM), and arrhythmogenic cardiomyopathy (ACM)—are among the most prevalent genetic cardiac disorders, with an estimated global prevalence of 0.2%. However, the true burden is likely underestimated due to incomplete penetrance, variable clinical expression, and historical emphasis on high-penetrance pedigrees in genetic studies.^1^ Although morphological classification continues to guide clinical decision-making, the diagnosis and management of inherited cardiomyopathies are hindered by genetic heterogeneity, overlapping mutations, and limitations in current diagnostic approaches.^2^, ^3^, ^4^

Artificial intelligence (AI), particularly machine learning (ML), deep learning (DL) and natural language processing (NLP), is advancing cardiology by enhancing the precision and efficiency of risk stratification, diagnosis, and prognosis in inherited cardiomyopathies. These techniques have demonstrated superior performance over conventional methods in interpreting electrocardiographic (ECG) and imaging data.^5^, ^6^, ^7^ However, current risk models are constrained by limited predictive accuracy, poor external validation, and reliance on static variables, with inadequate incorporation of clinical and genetic complexity. These limitations highlight the need for dynamic, multiparametric models that integrate genetic data and broader patient-specific factors to improve risk prediction.^8^, ^9^, ^10^, ^11^, ^12^

In this context, AI-based approaches hold significant promise for improving cardiovascular care in underserved settings by using accessible demographic, clinical, and imaging data which could enable early detection, guide personalized management, and help mitigate the burden of sudden cardiac death (SCD) in vulnerable populations.

## Current risk stratification tools and its limitations

Risk stratification in inherited cardiomyopathies remains clinically challenging, due to substantial genetic and phenotypic heterogeneity and the limitations of existing tools, which often lack accuracy, external validation, and integration of imaging, genetic, and longitudinal data.

### DCM

In DCM, risk stratification typically relies on static parameters such as left ventricular (LV) ejection fraction (EF), which often fail to capture temporal changes in disease severity and progression, thereby limiting long-term prognostic value.^13^

First-degree relatives of patients with DCM have an elevated risk of developing the condition, including SCD presentations. However, the familial screening has shown that up to 20% of relatives demonstrate features of DCM, even without a reported family history.^14^ Furthermore, genetic testing identifies pathogenic variants in only 40% of cases, with incomplete penetrance requiring ongoing surveillance via serial imaging.^15^ Although current guidelines recommend routine screening with ECG and echocardiography, implementation in clinical practice remains limited due to cost, resource constraints, and low uptake among asymptomatic individuals.^11^

### HCM

Although the European Society of Cardiology and the American College of Cardiology/American Heart Association SCD risk models are commonly used in HCM risk stratification, they have notable limitations. These include underestimating risk in pediatric patients, post-septal reduction cases, and underrepresented ethnic groups. Their reliance on maximal wall thickness introduces interobserver variability and potential misclassification, leading to unnecessary implantable cardioverter defibrillator (ICD) implantation in some cases.^16^, ^17^, ^18^, ^19^ Additionally, LV outflow tract obstruction, while recognized as a risk factor, does not fully reflect risk, particularly in Black patients, who often present with non-obstructive HCM, but exhibit extensive myocardial fibrosis on cardiac magnetic resonance (CMR)—an established predictor of adverse outcomes.^20^

### ACM

Risk stratification in ACM centers on balancing the benefit of ICD placement for SCD prevention against the risk of overtreatment. The 2010 Task Force Criteria established the first structured framework for arrhythmogenic right ventricular (RV) cardiomyopathy (ARVC) diagnosis, followed by refinement in the 2015 International Task Force Consensus.^21^^,^^22^ Further risk models, including the 2019 ARVC risk calculator, have incorporated clinical and imaging variables such as age, sex, syncope history, ventricular ectopy, T-wave inversions, and RVEF, offering improved stratification.^23^ However, they showed limited utility in gene-negative patients and those with left-dominant phenotypes.^24^^,^^25^ Similar to other cardiomyopathies, generalizability across diverse patient populations remains limited, reinforcing the need for more individualized and adaptable risk prediction tools.^26^

## AI models and their applications in cardiomyopathy risk stratification

Key AI technologies in cardiomyopathy risk stratification include ML, DL, and NLP. ML methods rely on manually selected features, while DL using convolutional and recurrent neural networks automatically extracts complex patterns from ECG and imaging data, and are often associated with superior performance due to its ability to model complex data without human input. In contrast, NLP enables automated extraction of relevant information from unstructured clinical texts, improving diagnostic efficiency and workflow.^27^^,^^28^

### Role of AI in cardiomyopathy risk stratification

#### Electrocardiogram

AI-ECG interpretation enables automated, highly accurate detection of different types of cardiomyopathies with high diagnostic performance. These models can identify subtle ECG patterns often missed by human readers, facilitating earlier diagnosis and risk assessment.^29^^,^^30^

#### Cardiac imaging and phenotyping

ML has been used to compare echocardiographic videos with automated measurements reducing interobserver variability. ML has also been applied to analyze 3-dimensional LV structures from CMR, enabling tree-based classification, a method that uses decision-tree algorithms to categorize data based on learned feature splits—for phenotype identification and personalized therapy.^31^ Additionally, ML-based analysis of late gadolinium enhancement (LGE) regions has shown potential in predicting ventricular tachycardia in patients with underlying cardiomyopathy.^32^ Furthermore, automated image segmentation of RV and LV structures on CMR images, can help to minimizes the need for manual input and improves the consistency of diagnostic interpretation.

#### Genomic variant interpretation

ML techniques enhance the interpretation of genetic variants by assigning probabilistic scores to variants of uncertain significance improving post-test diagnostics. Gene-specific and high-throughput models like the Learning from Evidence to Assess Pathogenicity model have demonstrated strong performance in predicting pathogenicity, thereby improving post-testing diagnostics and clinical variant classification.^33^, ^34^, ^35^ In addition, ML models that use tree-based algorithms have shown high accuracy in identifying pathogenic mutations and enhancing their clinical applicability.^36^

### Applications of AI in cardiomyopathy risk stratification

#### DCM

AI is increasingly being used to enhance risk stratification in DCM. By analyzing large datasets from imaging, clinical records, and genetic information, AI algorithms can identify subtle patterns and predict adverse outcomes, such as heart failure (HF) progression or arrhythmic events. This allows for more personalized treatment plans, early intervention, and improved patient management, ultimately leading to better prognostic accuracy and optimized care for individuals with DCM.^37^, ^38^, ^39^

##### AI DCM risk stratification models (summarized in Table 1)

###### ECG-based models in DCM

AI-enhanced ECG models have demonstrated high sensitivity (≥99%) and near-perfect negative predictive value (∼100%) in large cohorts, supporting their use as effective screening tools.^40^ These models maintained diagnostic accuracy across diverse ECG patterns, body types, and comorbidities, except in cases with left bundle branch block. Their high sensitivity enables efficient triaging, potentially reducing reliance on routine echocardiography and limiting unnecessary imaging in low-risk individuals.^4^

**Table 1:** Summary of AI models for DCM

Category	Model/study	Key features	Performance/impact
**ECG models**	Shrivastava et al, 2021; Rosenbaum et al, 2019	AI-enhanced ECG	High sensitivity (≥99%), cost-effective, limits routine echocardiography.
**Imaging models**	Mintz & Brodie, 2019; Yasaka et al, 2018	AI-driven tools	Enhances imaging accuracy and interpretation.
	Backhaus et al, 2019; Goyal et al, 2020	DL for LV measurement	Improves LV measurement and identification.
	Mariscal Harana et al, 2021	4D flow CMR and GANs	Enhances diagnostic precision.
	Moccia et al, 2019; Fahmy et al, 2019	FCNN for scar segmentation	Enhances scar segmentation and fibrosis quantification.
**Multimodal models**	R. Chen et al, 2019	ML integration	Integrates clinical data to predict cardiovascular events, surpassing LVEF-based systems.
	Peressutti et al, 2017; Cikes et al, 2019	ML for CRT prediction	Predicts CRT response effectively.
	Ameling et al, 2013; Ware et al, 2016	ML for imaging-genetics	Combines gene patterns with imaging for remodeling prediction.
	Aung et al, 2019	GWAS leveraging AI	Identifies genetic loci linked to heart failure risk.
	Kolk et al, 2024; Corianò et al, 2024	Multimodal AI using ECG, MRI, and clinical data for personalized arrhythmia risk prediction	Superior to single modalities; enhances diagnostic accuracy and individual risk stratification.

AI = artificial intelligence; CMR = cardiac magnetic resonance*;* CRT = cardiac resynchronization therapy DCM = dilated cardiomyopathy; DL = deep learning; *ECG =* electrocardiographic; FCNN = fully convolutional neural networks; GAN = generative adversarial network; GWAS = Genome Wide Association Study; LVEF = left ventricular ejection fraction; ML = machine learning; MRI = magnetic resonance imaging.

###### Imaging-based models in DCM

Advancements in AI have significantly enhanced the accuracy and reproducibility of cardiac imaging by improving image acquisition, segmentation, and analysis with minimal manual input.^41^^,^^42^ DL models have demonstrated high precision in LV contouring, scar segmentation and T1 mapping and volume quantification in CMR, reducing reliance on manual post-processing and enabling accurate delineation of myocardial fibrosis with reduced operator dependence, thus facilitating efficient, routine tissue characterization and more precise risk stratification in DCM.^43^, ^44^, ^45^, ^46^ Furthermore, emerging technologies such as 4-dimensional flow CMR and generative adversarial networks improved diagnostic accuracy further by characterizing altered flow dynamics and synthesizing high-quality CMR images across datasets.^47^^,^^48^

#### HCM

AI-based tools are increasingly applied in HCM to improve risk prediction by detecting high-risk features such as systolic dysfunction and myocardial fibrosis using non-invasive modalities like ECG and imaging.^49^ ML models have enhanced diagnostic accuracy in measurements such as maximal wall thickness and LGE, supporting more precise and individualized risk stratification through the integration of multiple phenotypic and disease-related variables.^50^^,^^51^

##### AI HCM risk stratification models (summarized in Table 2)

###### ECG-based models in HCM

Recent advances in DL have enabled the use of ECG data to identify high-risk features in HCM including systolic dysfunction, severe hypertrophy, apical aneurysms, and extensive LGE. Carrick et al^52^ developed a DL model using ECG data to identify high-risk features in HCM, achieving 97% sensitivity and, when combined with echocardiography, reducing the need for cardiac magnetic resonance imaging by 61%. Additional studies have associated specific ECG patterns—such as QRS and ST-T wave changes—with HF severity,^53^ while T-wave inversion has been recognized as a marker of elevated arrhythmic risk in patients with HCM.^54^

**Table 2:** Summary of AI models for HCM

Category	Model/study	Key features	Performance/impact
ECG models	Carrick et al, 2024^52^	DL for high-risk features (eg, LGE, hypertrophy).	97% sensitivity; reduced CMR by 61%.
	Togo et al, 2023^53^	DL: QRS/ST-T linked to HF severity.	Improved HF severity assessment.
	Lyon et al, 2018^54^	ML: T-wave inversion for arrhythmic risk.	Enhanced arrhythmic risk prediction.
Imaging models	Fahmy et al, 2021^55^	ML with echo/clinical data for HF progression.	80% sensitivity, 72% specificity.
	Mancio et al, 2021^56^	ML: Cine CMR for fibrosis prediction.	AUC 0.83; reduced gadolinium use.
	Augusto et al, 2021^50^	ML for precise LV wall thickness.	Refined SCD risk stratification.
	Navidi et al, 2023^51^	SAUNet: DL for LV scar via CMR LGE.	High accuracy; interpretable spatial maps.
Multimodal models	Smole et al, 2021^57^	HCM-RSS: ML for 5-year event prediction.	Robust ML-based risk prediction.
	Kochav et al, 2021^58^	Elastic net ML for event prediction.	Sensitivity: 88%, Specificity: 84%.
	Tower-Rader et al, 2020 (LVH-Fusion)^59^	Multimodal model integrating ECG and echo data.	Enhanced accuracy and interpretability.
	Lai et al, 2024 (MAARS-HCM)^60^	Combines clinical, symptomatic, imaging data for risk stratification.	Improved clinical management strategies
	Goto et al, 2022^61^	Federated learning with ECG and echo data across multiple institutions.	Accurate diagnosis; maintained data privacy
	Duffy et al, 2022 (EchoNet-LVH)^62^	DL on echo videos distinguishing types of hypertrophy.	High accuracy (AUC 0.98) in differentiating HCM.
	Peng et al, 2024^63^	DL with multi-view echo data to differentiate HCM and amyloidosis.	High diagnostic accuracy; enhanced versatility.

AI = artificial intelligence; AUC = area under the curve; CMR = cardiac magnetic resonance; DL = deep learning; ECG = electrocardiographic; HCM = hypertrophic cardiomyopathy; HCM-RSS = hypertrophic cardiomyopathy risk stratification system; HF = heart failure; LGE = late gadolinium enhancement; LV = left ventricular; LVH = left ventricular Hypertrophy; MAARS = Multimodal artificial intelligence for ventricular Arrhythmia Risk Stratification; ML = machine learning; SAUNet = Shape Attentive U-Net; SCD = sudden cardiac death.

###### Imaging-based models in HCM:

ML models have shown promise in enhancing risk assessment using imaging in HCM. An ML model created by Fahmy et al^55^ used echocardiographic and clinical data to predict HF progression, including LV outflow tract obstruction, symptom burden, and comorbidities, with a sensitivity of 80% and specificity of 72%. Mancio et al^56^ also developed another model that used cine CMR to estimate the likelihood of myocardial fibrosis, reducing the need for gadolinium contrast. ML has further improved measurement accuracy, with studies showing that automated wall thickness quantification reduces interobserver variability.^50^ Additionally, the Shape Attentive U-Net, a DL model for automated scar quantification, closely approximated expert manual analysis and produced interpretable spatial heatmaps for clinical decision support.^51^

#### ACM

AI is increasingly being applied to risk stratification in ACM, particularly for its subtype, ARVC. AI-driven models, including risk calculators, integrate data from electrocardiograms, cardiac imaging, and genetic profiles to predict the likelihood of arrhythmic events and guide decisions such as ICD placement.^64^

##### AI ACM risk stratification models (summarized in Table 3)

###### ECG-based models in ACM

ECG abnormalities, particularly precordial T-wave inversions, are critical markers in diagnosing ACM and predicting arrhythmic risk.^65^ Convolutional neural network and DL models have demonstrated high diagnostic accuracy for ACM, with a convolutional neural network model achieving 98% accuracy in distinguishing abnormal ECGs from normal ones.^66^ AI tools can also analyze fragmented QRS patterns linked to myocardial fibrosis and ventricular arrhythmia risk, potentially improving early diagnosis and risk stratification.^28^

**Table 3:** Summary of AI models for ACM

AI application	Key insights
**AI-ECG**	CNN/DL models achieve ∼98% accuracy in ACM diagnosis.^66^ AI can analyze fragmented QRS for fibrosis and VA risk.^28^^,^^67^
**AI-Echo**	AI-driven strain analysis links LV GLS and RV strain patterns to VA risk.^28^^,^^68^^,^^69^
**AI-CMR**	AI-based segmentation (99% accuracy) improves efficiency.^70^ AI can refine LGE-based risk prediction.^71^^,^^72^
**Genetics**	AI improves VUS classification and omics integration for better ACM diagnostics.^28^^,^^73^
Multimodal AI	Multimodal AI integrates various imaging and clinical data (LGE-CMR, ECG, clinical profiles, and myocardial thickness maps) significantly enhancing accuracy in ACM diagnosis and arrhythmia risk prediction.^74^, ^75^, ^76^

ACM = arrhythmogenic cardiomyopathy; AI = artificial intelligence; CMR = cardiac magnetic resonance; CNN = Convolutional neural network; DL = deep learning; ECG = electrocardiographic; GLS = global longitudinal strian; LGE = late gadolinium enhancement; LV = left ventricular; RV = right ventricular; VA = ventricular arrhythmia; VUS = variants of uncertain significance.

###### Imaging-based models in ACM:

AI-enhanced echocardiographic analysis, focusing on strain patterns, has shown strong correlation between RV strain abnormalities and ventricular arrhythmia incidence.^68^^,^^69^ AI-based models for CMR, such as a Bayesian dilated residual neural network, have demonstrated excellent accuracy in automated ventricular segmentation, reducing manual effort while maintaining diagnostic precision and achieving 99% accuracy in classification.^70^ Additionally, AI has been used to identify novel LGE patterns, aiding in distinguishing ACM from other cardiomyopathies like DCM and improving risk prediction.^71^

## Multimodal AI applications in risk stratification

Advancements in AI have enabled the integration of multimodal data—including imaging, ECG, clinical variables, genetic information, and biomarkers—into unified models that improve risk stratification across inherited cardiomyopathies. These approaches address the limitations of conventional tools by incorporating dynamic, individualized, and disease-specific features.

### DCM

ML and DL are increasingly used to combine clinical, imaging, and biomarker data for enhanced prediction of adverse outcomes in DCM.^77^ Chen et al^78^ developed an ML model that incorporated ECG, CMR, and baseline clinical variables which outperformed traditional scoring systems based solely on LVEF. Similarly, Zhou et al^79^ applied ML to longitudinal clinical and imaging data in oncology patients, achieving high accuracy in predicting HF and therapy-related cardiac dysfunction—highlighting its potential applicability in reversible forms of DCM.

AI models integrating data from ECG, echocardiography, and CMR, have also demonstrated strong predictive power for identifying cardiac resynchronization therapy responders and estimating LV volume reduction.^80^^,^^81^

In imaging-genetics applications, ML has been used to predict reverse remodeling following immunoadsorption therapy by linking myocardial gene expression—such as titin (TTN) mutations—with imaging biomarkers.^82^

Recent multimodal DL models, such as Kolk et al^74^’s integrative LGE-CMR, ECG, and clinical data model, have further improved arrhythmic risk prediction in non-ischemic HF. Similarly, The Deep ARrhythmic Prevention in DCM model, combined multidimensional CMR parameters with clinical variables to generate individualized arrhythmic risk curves to enable more personalized and accurate risk assessment in DCM management.^37^

### Multimodal AI models in HCM

Smole et al^57^ developed the HCM risk stratification system model, which integrates clinical, genetic, imaging, and historical data to predict 5-year risk of adverse events such as ventricular tachycardia, HF, and SCD using multiple ML algorithms demonstrating high accuracy in predicting events in patients with HCM.

Innovative models like LV Hypertrophy-Fusion (using ECG and echocardiographic data), EchoNet-LV Hypertrophy (using echocardiogram videos) and Multimodal AI for ventricular Arrhythmia Risk Stratification model (using clinical data, symptomatic evaluation, and cardiac imaging modalities) enhanced disease differentiation from hypertensive heart disease and arrhythmia risk stratification.^59^^,^^60^^,^^62^

Federated learning frameworks using ECG and echocardiographic data across institutions have accurately diagnosed HCM while preserving data privacy.^61^ Similarly, a multi-view DL model accurately differentiated HCM from cardiac amyloidosis, demonstrating the versatility of multimodal AI in diagnostic refinement.^63^

### Multimodal AI models in ACM

In ACM, multimodal AI has enabled the integration of clinical, imaging, genetic, and multi-omics data to improve diagnostic accuracy and individualized risk prediction. Furthermore, AI-based models have demonstrated high performance in classifying variants of uncertain significance and identifying key genes linked to disease severity such as PKP2, DSC2, DSG2, and DSP as well as novel variants.^28^ Additionally, multi-omics frameworks incorporating transcriptomic, proteomic, and metabolic data have uncovered new biomarkers and disease pathways.^73^

Similar to its application in DCM, Kolk et al^74^'s model demonstrated strong performance in ACM for predicting As by integrating LGE-CMR, ECG, and clinical data. Cine CMR combined with AI-based strain analysis has also accurately distinguished ACM from healthy individuals,^75^ while graph convolutional network models integrating computed tomography-derived myocardial thickness with clinical data have significantly improved arrhythmic risk prediction.^76^

## Limitations of AI in risk stratification of inherited cardiomyopathies

Despite its potential, the clinical adoption of AI-based tools in inherited cardiomyopathies still faces several challenges.

### Limited population diversity

Many AI models are developed using datasets that lack adequate representation of diverse populations which can lead to biased predictions and reduced generalizability, particularly in minority groups.^57^

### Overfitting and poor generalization

DL models are prone to overfitting when trained on small or homogeneous datasets, limiting their ability to generalize across diverse clinical populations and settings.^58^

### Interpretability and the “Black Box” problem

The "black box" nature of DL models where internal decision-making processes are not easily understood limits interpretability, reduces clinician trust, and hinders integration into clinical practice.^49^

## Data availability and privacy concerns

Limited access to large, diverse clinical datasets—along with data privacy concerns—reduces the accuracy, reliability, and generalizability of AI models.^49^

### Regulatory and implementation barriers

The lack of standardized regulatory frameworks for AI in health care hinders clinical validation and limits integration into routine practice.^49^

## Future directions of AI in risk stratification of inherited cardiomyopathies in under-resourced settings

AI holds significant potential to enhance diagnosis, risk stratification, and personalized treatment in inherited cardiomyopathies. Realizing this potential requires addressing current limitations related to data diversity, model transparency, and regulatory guidance. Expanding access to large, high-quality datasets—especially from underrepresented populations—is critical to improving model generalizability.

Federated learning offers a promising approach, enabling multi-institutional model development without compromising data privacy. Multicenter collaboration is also essential to support external validation and ensure consistency across AI-driven tools.^83^

Collaboration in data and model sharing will accelerate innovation and enable comparative evaluation of AI techniques. To support clinical integration, regulatory bodies must define clear validation standards and oversight mechanisms. Explainable AI is key to enhancing transparency and clinician trust by clarifying how predictions are generated, thereby increasing trust and facilitating adoption in clinical settings.^84^

**Figure 1:**
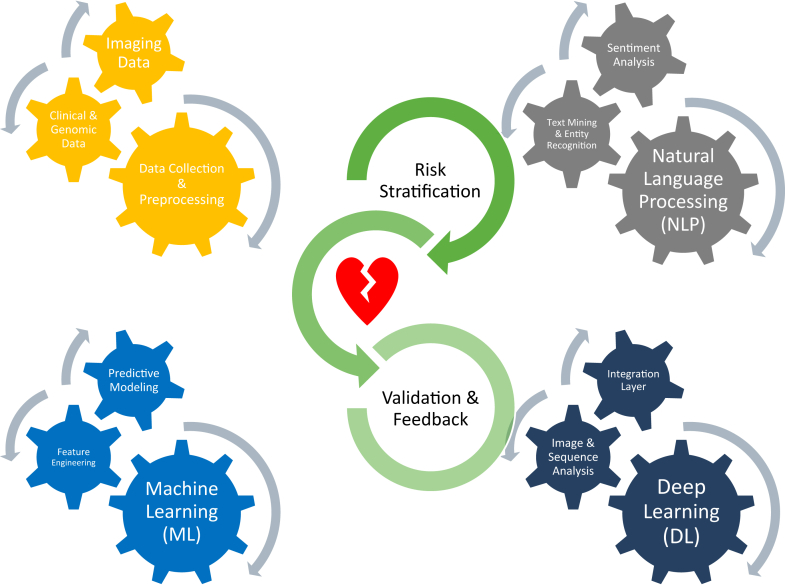
Integrative AI framework for risk stratification of inherited cardiomyopathies. AI = artificial intelligence.

Integrating multi-omics data—including genetic, transcriptomic, and proteomic information—with imaging and electrophysiology could further refine risk prediction models. A multidisciplinary approach involving clinicians, data scientists, and regulatory stakeholders is vital for protocol standardization, ethical oversight, and responsible AI deployment (Figure 1).

**Figure 2:**
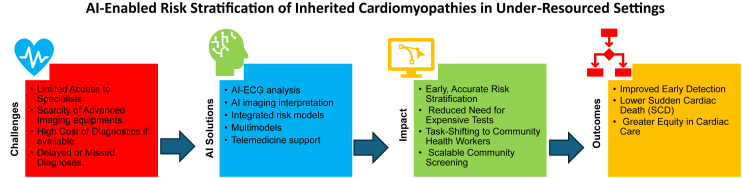
AI-enabled risk stratification of inherited cardiomyopathies in under-resourced settings. AI = artificial intelligence; ECG = electrocardiographic.

In under-resourced settings, AI has the potential to significantly improve cardiovascular care by providing scalable, cost-effective tools for risk stratification (Figure 2). AI-enhanced ECG models have demonstrated high accuracy in detecting LV systolic dysfunction and low EF from standard 12-lead ECGs, improving early diagnosis, and reducing reliance on imaging.^85^^,^^86^ Similarly, DL models applied to clinical notes have accurately identified patients with HF with reduced EF, streamlining quality assessment and adherence to guideline-based therapies.^87^

In DCM, AI-ECG models have shown strong sensitivity and negative predictive value, enabling simple and accessible screening.^40^ In HCM, DL-ECG models have identified high-risk features such as systolic dysfunction and extensive LGE with 97% sensitivity, reducing the need for CMR when used alongside echocardiography.^52^ Furthermore, the Screening for Peripartum Cardiomyopathies Using Artificial Intelligence (SPEC-AI) Nigeria trial further demonstrated that AI-guided screening significantly improved detection of peripartum cardiomyopathy.^88^

Despite these advances, investment in digital infrastructure and development of context-specific models are essential to ensure equitable and accurate deployment and overcome limited access to diagnostic tools, low digital literacy, and economic instability.

Table 4 summarizes proposed AI risk-stratification models applicable to under-resourced settings.

**Table 4:** Summary of AI models for AI in under-resourced settings

AI application	Model/study	Key insights
**AI-ECG for HFrEF**	Dhingra et al, 2025^85^; Yao et al, 2025^86^; Nargesi et al, 2025^87^	Detects LV dysfunction, improves early HF diagnosis, and enhances therapy implementation.
**AI-ECG for DCM**	Shrivastava et al, 2021^40^	High sensitivity, cost-effective screening tool.
**AI-risk in CCTA**	Tsiachristas et al, 2025^89^	Refines risk-guided management, improving outcomes.
**DL-ECG in HCM**	Carrick et al, 2024^6^	Identifies high-risk features with 97% sensitivity, reducing CMR need.

AI = artificial intelligence; CCTA = coronary computed tomography angiography; CMR = cardiac magnetic resonance; DCM = dilated cardiomyopathy; DL = deep learning; ECG = electrocardiographic; HFrEF = hear failure with reduced ejection fraction; LV = left ventricular.

## Conclusion

Risk stratification for SCD in inherited cardiomyopathies remains challenging due to genetic heterogeneity and limitations of conventional tools, particularly in resource-limited settings.

AI, especially ML, NLP and DL, offers promise by integrating multimodal data to enhance prediction and personalize care. However, barriers such as limited data diversity, lack of external validation, and interpretability concerns must be addressed.

By leveraging routine clinical data, automating complex analyses, using federated learning, explainable AI, and strengthened digital infrastructure, AI has the potential to improve early diagnosis, guide targeted interventions, and reduce the global burden of SCD, particularly in under-resourced populations.
